# Inflammation, Autoinflammation and Autoimmunity in Inflammatory Bowel Diseases

**DOI:** 10.3390/cimb45070350

**Published:** 2023-06-30

**Authors:** Andrea Padoan, Giulia Musso, Nicole Contran, Daniela Basso

**Affiliations:** Department of Medicine—DIMED, University of Padova, Via Giustiniani 2, 35128 Padova, Italy; andrea.padoan@unipd.it (A.P.); nicole.contran@phd.unipd.it (N.C.); daniela.basso@unipd.it (D.B.)

**Keywords:** inflammatory bowel diseases, autoimmunity, cytokines, autoinflammation

## Abstract

In this review, the role of innate and adaptive immunity in the pathogenesis of inflammatory bowel diseases (IBD) is reported. In IBD, an altered innate immunity is often found, with increased Th17 and decreased Treg cells infiltrating the intestinal mucosa. An associated increase in inflammatory cytokines, such as IL-1 and TNF-α, and a decrease in anti-inflammatory cytokines, such as IL-10, concur in favoring the persistent inflammation of the gut mucosa. Autoinflammation is highlighted with insights in the role of inflammasomes, which activation by exogenous or endogenous triggers might be favored by mutations of NOD and NLRP proteins. Autoimmunity mechanisms also take place in IBD pathogenesis and in this context of a persistent immune stimulation by bacterial antigens and antigens derived from intestinal cells degradation, the adaptive immune response takes place and results in antibodies and autoantibodies production, a frequent finding in these diseases. Inflammation, autoinflammation and autoimmunity concur in altering the mucus layer and enhancing intestinal permeability, which sustains the vicious cycle of further mucosal inflammation.

## 1. Introduction

Inflammatory bowel diseases (IBD), comprising Crohn’s disease (CD) and ulcerative colitis (UC), which typically occur in young adults and children, are persistent with chronic relapses and remittance. The prevalence of IBD, estimated at 0.6 to 1% in industrialized countries, has increased worldwide, from 3.7 million cases in 1990 to 6.8 million in 2017, increasing in particular in regions with historically low rates and limited resources, probably due to the spread of Westernized lifestyle and dietary habits [1]. IBD mainly affect the gastrointestinal tract, UC involving the colon mucosa and CD causing transmural inflammation in any part of the gastrointestinal tract, from the mouth to the anus. However, extra-intestinal manifestations involving the skin, the musculoskeletal system, the eyes and other organs are not infrequent. Clinically, their onset might be insidious with unspecific symptoms often mimicking those of functional diseases, such as irritable bowel syndrome (IBS), or hyperacute with bloody diarrhea, weight loss and abdominal pain. These diseases persist lifelong, the clinical course varying from patient to patient, sometimes with persistent remission or, more frequently, with alternating remission and flares. The various conditions potentially triggering flareups, include infections, stress events, environmental factors and drugs. The pathogenesis of these diseases is not yet fully understood, although an altered innate and adaptive immune response associated with the disequilibrium of the intestinal microbiome with genetic predisposition appears to be the most likely hypothesis. Chronic inflammation that characterizes IBD appears to involve the inflammasome and the autophagy pathways as well as inflammatory cells and cytokines and is often associated with autoimmunity. The pathogenesis of autoimmune diseases is characterized by loss of tolerance against self-antigens and by the production of auto-antibodies detectable in blood with involvement of adaptive immunity. Autoinflammatory diseases share with autoimmune diseases several clinical signs, but they are mainly due to altered innate immunity and activation of the inflammasomes by exogenous and/or endogenous triggers, without auto-antibodies production. In IBD, both autoimmunity and autoinflammation co-exist.

## 2. Inflammation in IBD

The comprehensive view of IBD pathogenesis takes into account several factors, including the complex interplay between the patient’s genome with the “exposome” and the “immunome” [2]. More than 200 IBD-associated genetic loci have been reported [3], which account for about one-fourth of all cases and involve genes associated with inflammation and autophagy [2,4,5,6].

**Figure 1:**
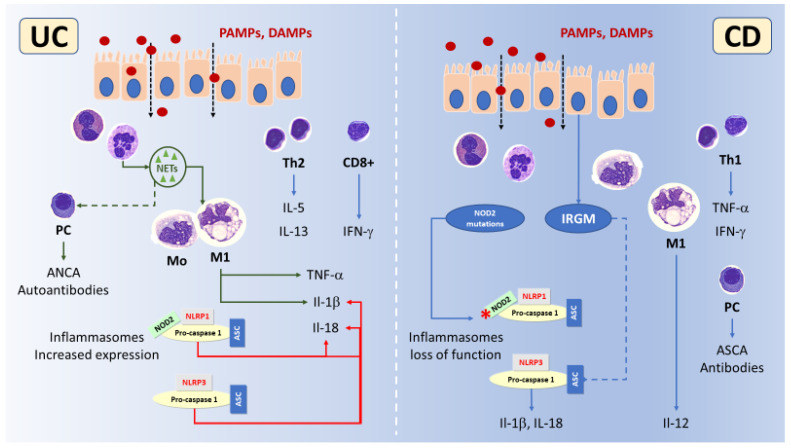
Schematic illustration of the main differences between ulcerative colitis (UC, **left**) and Crohn’s disease (CD, **right**) in inflammation, autoinflammation and autoimmunity. The increased intestinal permeability of the epithelial cell layer allows in both diseases Danger (DAMPs) and Pathogen (PAMPs) associated molecular patterns (red dots) to easily reach the mucosa and stimulate innate immune cells. In UC neutrophil extracellular traps (NETs, green) are more abundant than in CD, activate M1 macrophages inducing TNF-α and IL-1β release. It is possible that NETs activate also the adaptive immune response causing ANCA autoantibodies production. The expression of inflammasomes, mainly NLRP1 and NLRP3, is induced in UC concurring in further enhancing IL-1β. CD4+ Th2 and CD8+ cells with IL-5, IL-13 and IFN-γ production prevails. In CD CD4+ Th1 cells prevails over Th2 with TNF-α and IFN-γ production. M1 macrophages produce mainly IL-12, while loss of function of the inflammasomes NLRP1 and NLRP3, due to NOD2 mutations and increased Immunity-related GTPase M (IRGM) that enhances NLRP3 degradation, occurs.

Any environmental factor in which a subject might be exposed is included in the collective noun “exposome”, where the endogenous components are represented by the microbiome [2]. Following exposure, the intestinal mucosal immune reaction (“immunome”) leads to inflammation and tissue damage accompanied by innate and adaptive immune response, with a predominant Th1 and Th17 response with IL-12, interferon (IFN)-γ and IL-17A production in CD [7] and a Th2 response with IL-5 and IL-13 production in UC (Figure 1) [2,8]. Intestinal inflammation is often associated with intestinal barrier alterations due to decreased mucin gene expression and/or altered tight-junction (TJs), resulting in increased permeability that may trigger activation of dendritic cells (DCs) in the lamina propria through Toll-like (TLR) and nucleotide binding domain-like receptors (NLR) by the enteric microflora antigens [9,10,11]. Other immune cells involved in IBD-associated intestinal inflammation are natural killer (NK) cells and innate immune cells derived from lymphoid progenitors (ILCs) group 3 producing the Th17 cell-associated cytokines IL-17 and IL-22 [12,13].

### 2.1. Intestinal Barrier Alterations

#### 2.1.1. The Mucus Layer

The intestinal epithelial cells (IECs), which are linked to each other by the TJs, mainly made up of claudins, occludins and F-actin, are protected by a dense mucus layer. In addition to mucus, mucin glycoproteins (MUC), defensins, immunoglobulins and antimicrobial peptides constitute the first line defense of IECs from the harmful lumen contents. Other specialized cells, such as Paneth cells, goblet cells and stem cells, concur in the epithelium together with immune cells. Intestinal health also depends on commensal bacteria, the largest microbiome in the human body, and their prevalence in the colon being particularly elevated [14].

The mucus layer lubricates the passage of stool along the intestine, but is also a barrier, the primary physical defense of the host from microbiota and noxious agents, able also to limit the presentation of antigens [15]. The protective effect of mucus is important not only in preventing IBD, but also in limiting the risk of any subsequent neoplastic transformation [16]. The main components of mucus, Gel-forming MUC, are produced by Goblet cells, and can be subdivided into two major subtypes, namely, transmembrane and secreted mucins. Transmembrane MUC, such as MUC2, MUC3, MUC4, MUC12, MUC13 and MUC17, which are constitutively expressed in the gastrointestinal tract, form the glycocalyx [17], being MUC2 the predominant structural component of the mucus layer. Interestingly, a reduced synthesis of MUC2 has been observed in human and animal IBD models [17]. Accordingly, we previously found decreased MUC2 in CD, not in UC, stool with respect to controls [18]. Intestinal microbes by means of the local release of bioactive factors and the activation of different signaling cascades, might directly affect Goblet cell function and mucin production [19]. For example, lipopolysaccharides (LPS) and flagellin A from Gram-negative bacteria are the most common modulator of mucin production, affecting MUC2 and MUC5AC.

Within the mucin layer, immunoglobulins and secreted antimicrobial peptides such as defensins can be found. They play an important role in controlling microbial diversity and antigen penetration across the gut mucosa [20]. β-defensins are decreased in the mucus layer in UC and in colonic CD [21], and the correct expression of α-defensins appears crucial in preventing CD [22]. The release of α-defensins (HD5 and HD6) is provided by Paneth cell secretion, stimulated by cholinergic stimuli and bacterial factors through NOD2 activation of the NF-κβ pathway [23]. Defective Paneth cell differentiation in CD, linked to the absence of α-defensins, has been associated with an impaired mucosal barrier, allowing luminal microbes to invade the mucosa and trigger a secondary inflammatory response [21,24]. Neutrophil defensin 1 acts against a broad spectrum of infectious agents, promotes NLRP3 inflammasome and IL-1β release [25]. We have previously found that levels of this protein are significantly increased in CD, but mainly in UC stool as compared to controls, suggesting its potential role in disease pathogenesis and diagnosis [18].

#### 2.1.2. Secretory Immunoglobulins

Secretory IgA (sIgA) are the first arm of the mucosal immune system to limit pathogens, prevent imbalance and maintain homeostasis between commensals and pathogens microorganisms on the mucosal surface. This dimeric immunoglobulin class, which acts in situ [26], is able to neutralize not only pathogens but also toxins, such as Clostridium difficile toxin A. The sIgA-antigen immunocomplex can be internalized by Peyer’s patch M cells in the subepithelial dome region, and the bacterial components presented to tolerogenic DCs. This process aids in limiting the inflammation that otherwise could occur in the presence of the enormous quantity of bacteria present in the intestinal lumen [27]. However, in some pathological conditions, the abnormal apical-to-basal retro transport of sIgA immunocomplexes can mediate the entry of noxious antigens into the IECs [21]. In patients with IgA deficiency, secretory IgM and, to a lesser extent, secretory IgG, are generally presumed to compensate for lack of sIgA. However, it has been postulated that sIgA deficiency can cause a dramatic increase in pathobionts (commensal bacteria potentially harmful to host homeostasis under certain conditions) and may be associated with the increase in proinflammatory cytokines [28]. In a large cohort of IBD patients and controls, it has been demonstrated that 43 bacterial taxa were highly coated with sIgA and, in addition, immuno-therapy changes the microbiota-specific IgA responses with respect to controls [29]. The role of sIgA in IBD has been comprehensively reviewed by Bamias et al. [30].

#### 2.1.3. Tight Junctions Alterations

Alterations of the TJs are hallmarks of IBD, being both consequence and cause of the intestinal inflammation. In IBD, the expression of proteins entering in the TJs complex might be increased (e.g., pore-forming claudin 2) or reduced (e.g., pore-sealing claudin 4 and 5) [31,32].

The hypothesis that alterations of the TJs are a primary defect in IBD is supported by the observation that they can be found in IBD unaffected relatives, but also because they are reported as an early event that promotes disease initiation in animal models [32,33,34].

On the other hand, inflammatory cytokines have been demonstrated to induce pore-forming claudin 2 (IL-6 and IL-13) or alter occludin and ZO-1 expression (IL-1 and IFN-γ) [35,36,37].

Inflammatory associated matrix metalloproteinases and the extracellular neutrophil traps (NETs) also appear involved in altering TJs proteins causing increased intestinal permeability and supporting the hypothesis that alterations of the TJs are a consequence of inflammation [38,39,40].

In any case, the persistent inflammation in IBD sustains a vicious cycle with alterations of the TJs that lead to increased intestinal permeability, which might favor paracellular entry of pathogen-associated molecular patterns (PAMPs), but also of other molecules and/or chemicals from the environment and diet, each of which is a potential activator of TLRs and NOD-like receptors (NLR), ultimately causing inflammation [41]. This hypothesis is supported by the finding that in IBD patients, increased intestinal permeability is associated with ongoing bowel symptoms and increased severity of diarrhea [41,42]. Another consequence of “leaky gut” might be intestinal dysbiosis, also frequently associated with IBD [43,44].

#### 2.1.4. Innate Immune Receptors

The altered mucus layer and increased intestinal permeability favors microbial antigens recognition by innate immune receptors which further trigger inflammation. Several innate immune receptors are involved in microbial recognition in the superficial IECs, and these include Toll-Like (TLR), RIG-like (RLR), NOD-like and C-type lectin receptors. These receptors recognize evolutionarily conserved molecular structures, which are frequently referred to as microbial- or pathogen-associated molecular patterns (MAMPs or PAMPs) [20]. Different TLRs might be expressed by the same cell type and the same cell type might express different TLRs, any of them being engaged by specific MAMPs or PAMPs ligands. TLRs signaling results mainly in inflammatory pathways, such as NF-kB, which ultimately induce inflammatory cytokines. In IBD alterations of TLRs, expression occur as detailed in Table 1 [45,46,47]. Those TLRs with protective effects are commonly found to be reduced in the human mucosa and/or in animal models (e.g., TLR5, which also promotes Treg differentiation), while those promoting inflammation usually increase and might return to baseline values during remission, such as TLR2 and TLR3 [48,49,50,51,52,53,54].

#### 2.1.5. Laboratory Testing in IBD: Evaluation of Intestinal Barrier Integrity

Evaluation of intestinal barrier integrity can be performed with invasive procedures such as histology or confocal laser endomicroscopy (CLE) [42]. In clinical practice, small intestine permeability can be evaluated using the non-invasive lactulose-mannitol (L/M) test, which is sensitive and specific, allowing organic to be distinguished from functional diseases [41,55]. The L/M test is based on the oral administration of the monosaccharide mannitol and the di-saccharide, lactulose. Mannitol passes the intestinal epithelial barrier through a transcellular pathway (about 30%), while only traces (about 0.3%) of lactulose are normally absorbed and excreted [56], their measurement in 6-h collected urine allowing a comparison to be made between their excreted fractions, yielding an L/M ratio in healthy individuals of less than 0.02 [55].

### 2.2. Innate Immune Cells in IBD

Once the epithelial defenses are damaged or impaired, an increased flow of MAMPs from the lumen leads to the activation of the membrane receptors expressed by immune cells [16]. Innate immune cells, such as macrophages, innate lymphoid cells, mast cells and neutrophils, are deputed to rapidly identify and eliminate MAMPs, although their mechanisms are based on innate immune receptors, which recognize a limited number of molecules [57]. Antigen-presenting cells (APCs), macrophages and dendritic cells (DCs) gather the mechanism of innate and adaptive immune response at both the local site and at the peripheral lymph nodes [58].

#### 2.2.1. Neutrophils and NETs

The inflamed intestinal mucosa of IBD patients is particularly enriched in neutrophils, especially when flares occur. The intestinal neutrophilic inflammation is associated with neutrophils activation and release of inflammatory molecules including calprotectin, a well-established biomarker for diagnosing and monitoring IBD [18,59,60]. Neutrophils are also emerging as primarily involved in UC pathogenesis, through neutrophil extracellular traps (NETs), a network of extracellular fibers comprising decondensated chromatin, DNA and antimicrobial peptides that could express and transfer molecules and mediators (Figure 1). In UC infiltrating neutrophils, an increased expression of the Peptidyl arginine deiminase 4 (PAD4) enzyme, a driver of the release of NETs, has been described in both human and in animal model specimens [61,62]. In active UC, not in CD, NETs carrying IL-1β and tissue factor accumulate in the inflamed mucosa, being induced by REDD1 driven authophagy [63]. Moreover, NETs are stimulants for IL-1b and tumor necrosis factor (TNF-α) release by lamina propria mononuclear cells, being treated with anakinra, an IL-1b inhibitor, or with streptonigrin, an inhibitor of NETs formation, effective in reducing colonic inflammation [61,63].

#### 2.2.2. Dendritic Cells and Macrophages

In normal conditions, intestinal DCs are immune tolerant, secreting protective anti-inflammatory IL-10, whilst in IBD the number of pro-inflammatory DCs increases. Similarly, during inflammation activated macrophages secrete large amounts of pro-inflammatory cytokines, differently from macrophages performing normal phagocytic functions. Two activated macrophage types have been characterized: type M1, pro-inflammatory, and type M2, anti-inflammatory [58]. M1 macrophages, which present pro-inflammatory functions and have an antibacterial effect, are activated by INF-γ or GM-CSF. M2 macrophages, induced by IL-4, IL-10, IL-13 contribute to tissue healing and fibrosis [58]. In experimental and human IBD, M1 prevails over M2 macrophages, and molecules able to invert this trend, such as baicalin, IL-33, lactic acid bacteria or plant derived flavonoids, allow for the reduction of the severity of intestinal inflammation promoting mucosal healing, partly through the activation of Wnt signaling [64,65,66,67,68,69,70,71,72].

Mesenchymal stem cells (MSC) and their derived products including exosomes and tumor necrosis factor-α-induced gene/protein 6 (TSG-6) appear also able to induce M2 macrophages polarization, thus protecting intestinal mucosa from inflammation and being effective in IBD treatment. The efficacy of MSC on macrophages M2 polarization is enhanced when these cells express HIF-1a, which occurs when these cells are maintained in hypoxic niches, their naturally occurring home [73,74,75,76,77,78,79,80,81,82].

### 2.3. Adaptive Immune Cells in IBD

In a recent study evaluating the landscape of relative fractions of immune cell populations in IBD, it was shown that the proportion of adaptive immune cells was decreased in CD and UC in ileum, colon and rectum with respect to healthy controls, while the innate immune cells proportion was increased [83]. Furthermore, the relative fractions of the B- and T-cell populations were both decreased in IBD, showing that in subjects with this condition with respect to healthy individuals, there is an imbalance in both adaptive and innate immunity [83].

The adaptive immune cells preserve immune tolerance to intestinal microbiome by the constant crosstalk between cells and microbial antigens [84]. In IBD, adaptive immunity is mainly led by CD4+ and CD8+ T cells, and regulatory T cells (Treg), and the active participation of the different populations of cells accounts for heterogeneous effects and disease manifestation in IBD patients [85].

#### 2.3.1. CD4+ T Helper Cells

CD4+ T cells that comprise helper T (Th) and regulatory T cells (Treg) [16] have a pivotal role in IBD. Th cells’ subtypes exert different effects in IBD, with Th1 mainly involved in CD favoring intestinal inflammation and Th2 in UC (Figure 1) [16,85]. Th1 cells through the production of TNF-α and IFN-γ also activate cytotoxic CD8+ T cells. In IBD, a linking has been found between Th17 and Th1 cells, and Tregs impairment. Th17, pro-inflammatory T helper cells that evolve from CD4+ T cells in the presence of IL-6, transforming growth factor β (TGF-β), IL-21, express the receptor for IL-23, a cytokine required for their survival and proliferation. Th17 cells are characterized by the production of both IFN-γ and IL-17A, which dysregulated; excessive production occurs in the presence of immune system impairment [85], one of the important characteristics of IBD in the development of inflamed intestinal tissue. This feature is also common to other conditions, such as rheumatoid arthritis (RA) and psoriatic skin lesions [85].

Th2 cells exert different effects related to different activation states. Basically, they maintain mucosal homeostasis by regulating inflammation and tissue repair, also providing response to parasites [16,85]; but when Th2 cells are excessively activated, they may favor chronic inflammation. Th2 release several cytokines, some of which (e.g., IL-4 and IL-10) with a direct anti-inflammatory effect, while others (e.g., IL-5, IL-13, IL-21 and IL-25) reduce inflammation by dampening Th1 activation [16,85]. High levels of Th2-related cytokines, such as IL-5 and IL-13, have been described in the immune cells of UC patients. IL-5 plays a role in eosinophil differentiation, but the role of the latter cells in IBD is not yet fully understood [85]. Other Th cells, such as Th9, Th17 and Th22, play a particular role in the pathogenesis of UC, and often a higher number of these cells are found in biopsies from UC patients [16,85].

#### 2.3.2. CD8+ T Cells

In IBD mucosal inflammatory infiltrate, CD8+ T cells are also found, but their role in pathogenesis is not yet completely understood. In UC, these cells are probably involved in favoring inflammation and epithelial damage, ultimately leading to ulcer formation, through the release of pro-inflammatory cytokines such as IFN-γ and TNF-α [58]. In CD, their role is even more unclear and debated [86]. CD8+ T cells can assume different phenotypes depending on cytokine stimulation, co-stimulatory molecules, as well as the strength of the TCR/antigen engagement, and can finally differentiate into cytotoxic killer cells, which have been suggested as a major mediator of autoimmunity in IBD [86]. This hypothesis is supported by findings from Lee et al., who have demonstrated that CD8+ T cell transcriptomes are able to evidence patients with clinically diverging outcomes [87].

#### 2.3.3. CD4+ Treg Cells

Tregs, which consist of CD4+CD25+FOXP3+ lymphocytes, with heterogeneous subtypes of CD49-T cells exhibiting immunosuppressive properties [85], prevent the development of autoimmune processes since they secrete anti-inflammatory cytokines such as IL-10, IL-35 and TGF-β. The lack of IL-10 secretion by the Treg cells has been shown to cause spontaneous colitis in a mouse model [58]. In IBD patients, increased mucosal and decreased circulating Tregs could be generally found, accompanied by higher amounts of peripheral Th17 cells. The unbalance between pro-inflammatory and anti-inflammatory cells in IBD is further supported by the observation that in front of increased FOXP3 expression, increased levels of cytokines, such as IL-17A, IL-1 and IL-6, are also found in the inflamed mucosa [85].

#### 2.3.4. B-Lymphocytes

A central role in adaptive immunity is carried by B lymphocytes, which are also involved in IBD. B cells, which migrate to the intestinal mucosa from mesenteric lymph nodes, differentiate in plasmocytes in the lamina propria. In addition, the synthesis of antibodies and T cells antigen presentation, B lymphocytes secrete several cytokines, including IL-2, IL-4, and IFN-γ, TGF-β and GM-CSF, regulating inflammation [58]. Similarly to CD4+ T cells, B cells comprise a regulatory subset with immunosuppressive effects, which has been described to be decreased in UC patients [29,85].

### 2.4. The Role of Cytokines in IBD

Cytokines networks can moderate the cross-talk of epithelial cells with innate and adaptive immunity, the amount and nature of interaction can change over time [88]. Moreover, the understanding gained of cytokines networks is of fundamental importance in the development of biological therapies, which are transforming the treatment of IBD.

Microbial sensing plays a key role in cytokine production by both the immune and intestinal cells. Following their activation though TLR and NOD, DCs and macrophages produce a large amount of pro-inflammatory cytokines, such as IL-1β and IL-23, which in turn may activate Th17 cells [89]. Th17 and other cells, such as type 3 innate lymphoid cells (ILC3) [89], produce IL-22 that primarily acts on IECs, activating STAT3 to promote antimicrobial defense, barrier integrity and repair. Furthermore, a significant increase in IL-1 tissue levels has been found in UC patients with respect to those with other gastrointestinal symptoms [90]. The two cytokines, IL-2 and TGF-β, support the maintenance of intestinal immune regulation. They are produced by and act on CD4+ T cells, but they also act on Tregs. IL-6 might activate multiple targets such as APCs and T cells and stimulate the proliferation and expansion of IECs [91], thus stabilizing intestinal homeostasis. TNF-α, increased in the IBD inflamed mucosa, has a relevant pathogenetic role as supported by the clinical response to anti-TNF-α drugs. TNF-α, produced by several cell types, such as macrophages, adipocytes, fibroblasts and T cells, determine inflammation or cell death depending on the alternative engagement of its putative receptors followed by NF-kB or the apoptosis pathways activation, respectively [91]. A dysregulation in its levels, together with the increase of IL-17, IL-21, IL-22 and IL-9, can lead to IBD [92]. IL-17 expression, not detected in samples from healthy colonic mucosa, infectious colitis or ischemic colitis have been clearly identified in the inflamed mucosa of UC and CD patients [93]. Likewise, TNF-α expression has been found in colonic tissues and macrophages in both CD and UC [94], and both TNF-α receptor I and II levels have been correlated with disease activity in IBD patients. Furthermore, findings made in clinical studies have demonstrated an important improvement in CD patients following the administration of anti-TNF-α therapy, including infliximab, adalimumab and certolizumab [94]. IL-12 family heterodimeric cytokines (such as IL-12, IL-23, IL-27 and IL-35) are released by APCs during intestinal inflammation. Both DCs and macrophages present an increased production of IL-12 in CD, but not in UC. In turn, Th1 cells can produce IL-23, which perpetuates the Th17 response and downregulates the Treg response [91]. Th2, as well as other immune cell types such as natural killer T cells, produce IL-13, which increased the expression in the gut of IBD patients and appears involved in favoring fibrosis, one of the hallmarks of IBD-associated inflammation underlying the development of bowel stenosis [95].

IL-10 and TGF-β play a role in down-regulating inflammatory responses caused by continuous exposure to microbial products [92]. It has been demonstrated that, due to SMAD7 up-regulation, intestinal myeloid cells from IBD patients reduce responsivity to anti-inflammatory cytokines, including IL-10 and TGF-β. A decrease in TGF-β is believed to be responsible for the development of autoimmune disorders, including IBD [92]. Table 2 summarizes the characteristics and potential function of the cytokines with a major role in IBD.

## 3. Autoinflammation in IBD

Autoinflammation is increasingly recognized as a pathophysiological mechanism leading to IBD, as previously observed in reviews by Chen and Núñez and by Opipari and Franchi [97,98], this concept being based on clinical observations and findings in experimental studies.

### 3.1. Clinical Studies

From a clinical viewpoint, some extra-intestinal manifestations of IBD have been recognized as autoinflammatory diseases and, on the other hand, abdominal symptoms with intestinal inflammation occur in classical autoinflammatory diseases [5,99].

The first description of autoinflammatory diseases was provided by the International Familial Mediterranean Fever (FMF) consortium in 1997 following the discovery of missense FMF gene mutations (MEFV) in FMF individuals [100,101]. TNRSF1A, NLRP3 and MVK gene mutations are associated with three other hereditary recurrent fevers, namely TNF-α receptor-associated periodic syndrome (TRAPS), cryopyrin-associated periodic syndromes (CAPS) and mevalonate kinase deficiency [102]. In addition to the above-mentioned “four historical” autoinflammatory diseases, in the last two decades further studies have reported an increasing number of diseases ascribed to an autoinflammatory pathogenesis [103].

The panorama of autoinflammation in IBD has recently been extended by Tyler et al. [104], who described the novel DEX (deficiency in ELF4 X-linked) autoinflammatory disease in three unrelated male children. In addition to fever and oral ulcers, an IBD-like intestinal inflammation was described with neutrophil infiltrates, elevated expression of IL-17 in the ascending colon and a pro-inflammatory response of macrophages. The authors demonstrated that a variant of ELF4 transcription factor fails to promote anti-inflammatory genes, including IL-10 and IL-1RN, and favors pro-inflammatory gene transcription, mainly, IL-1, IL-23, IL-6 and CXCL1, upon stimulation with PRR ligands, including the NOD2 agonist, muramyl dipeptide.

### 3.2. Danger Signals and Receptors

In autoinflammatory diseases, the deregulated innate immune response to exogenous or endogenous danger signals triggers activation of the inflammasome, which typically determines the overactivation and release of IL-1 and IL-18 [105]. Autoinflammatory triggers include signals from damaged cells (danger associated molecular patterns—DAMPs), pathogen derived molecules, such as PAMPs, LPS and unmethylated CpG DNA, but also metabolites, such as glucose, free fatty acids, oxidized low-density lipoprotein (oxLDL), cholesterol crystals, uric acid crystals, ceramide, amyloid-β, α-synuclein and prion protein or superoxide dismutase [105,106]. PAMPs are recognized by the transmembrane and intracellular Pattern Recognition Receptors (PRRs), prototypes of the former and of the latter being the TLRs and the NOD-like receptor (NLR) family respectively [107]. The molecular structure of NLR is characterized by three domains: (1) leucin rich repeats (LRR) at the C-terminal, (2) nucleotide-binding and oligomerization (NOD) central, and (3) Pyrin (PYD) or caspase recruitment (CARD) at the N-terminal. PYD and CARD recruit and interact with binding partners such as the adaptor protein apoptosis-associated speck-like protein containing CARD (ASC) and caspase 1, which activates the pro-inflammatory IL-1 and IL-18 [107]. Once activated, NLR undergo both homo- and heterodimerization by binding with molecular partners to form large molecular platforms that can activate downstream effector molecules. Depending on the structural proteins that oligomerize, molecular platforms include the inflammasome, which promotes caspase activation, and the NODosome, which promotes NF-kB signaling [105,107]. An extensive cross-talk takes place between the inflammasome and the NODosome with the apoptosome, a complex involving apoptotic protease activating factor-1 (APAF-1), cytochrome c and caspase-9 [107]. Five families of NLR proteins have been described: NLR, NLRB, NLRC, NLRP and NLRX, those potentially involved in IBD being reported in Table 3. NLRP proteins comprise 14 members, their respective genes being named NLRP1 to NLRP14 [108]. The inflammasomes are defined on the basis of the NLRP proteins in the complex, resulting in more than 20 species, including the NLRP1, NLRP3 and NLRC4 inflammasomes as reported in Table 3.

### 3.3. NOD2 and IBD

The most striking association between IBD and NLR is that between CD and NOD2, but emerging evidence also highlights the association of IBD with NOD1 and NLRP3 [109]. The NOD2 gene encodes a protein with two CARD domains and six LRRs, the protein being primarily expressed in peripheral blood leukocytes. NOD2, stimulated in the intestine mainly by bacterial muramyl dipeptide (MDP), a fragment of bacterial peptidoglycan, interacts with the downstream signal molecule receptor-interacting serine/threonine kinase 2 (RIPK2) which recruits ubiquitin ligases and activates MAP kinases and NF-kB pathways [110,111], ultimately determining the production of proinflammatory cytokines and chemokines. The NOD2/RIPK2 complex also activates antibacterial autophagy, which limits intracellular bacteria proliferation, by means of the autophagy related protein 16-1 (ATG16L1) and the ubiquitin ligase X-linked inhibitor of apoptosis (XIAP) [112]. The association between CD and NOD2 and ATG16L1 mutations has been well-established, while no such association has been found for RIPK2 genetic variants. A frameshift mutation (L1007fsinsC) that determines a truncated LRR and a number of point mutations within the LRR of NOD2, mainly, Arg702Trp and Gly908Arg, is associated with NOD2 loss of function in CD, while other mutations associated with gain of function and constitutive NF-kB activation are recorded in Blau Syndrome and early onset sarcoidosis [107]. While loss of NOD2 function is known to impair bacterial sensing, it is not yet clear how it might determine inflammation. However, it is known that, in the presence of CD-associated NOD2 mutations, the intestinal epithelial barrier is compromised, probably as a consequence of an altered function of Paneth cells in secreting defensins.

### 3.4. NLRP Inflammasomes and IBD

NOD2 is involved in NLRP1 inflammasome activation and, in young IBD patients, NLRP1 polymorphism rs12150220 (Leu155His) is associated with resistance to steroids [113]. Intriguingly, increased NLRP1 expression found in the inflamed colon of UC patients, is positively correlated with IFN-γ gene expression and negatively correlated with the abundance of the Clostridiales bacterial species, which are suggested to have an anti-inflammatory effect in IBD [114], thus further supporting the presence of complex relationships between the microbiome and the inflammasomes. The NLRP3 rs6672995 and rs4353135 CD risk alleles are associated with decreased LPS-induced IL-1 production, and with a decrease in baseline NLRP3 expression, respectively, unlike gain-of-function mutations associated with the NLRP3 hereditary periodic fever syndromes [115]. However, increased NLRP3 mRNA expression levels have been found in ulcerated CD mucosa with respect to controls [115]. Another CD-associated risk factor, immunity-related GTPase M (IRGM) is functionally correlated with the NLRP3 [116,117]. Recently, IRGM was demonstrated to block NLRP3 assembly and oligomerization and to mediate NLRP3 autophagic degradation [118]. Again, in CD the reduction in inflammasome activity appears to take place and might, by reducing IL-18 release, alter the microbial composition and disrupt the epithelial barrier, thus favoring bacterial invasion and inflammation since IL-18 prevents epithelial damage, promotes Treg and limits Th17 expansion [119].

In UC, NLRP3 also appears to be involved through its interaction with NLRC4 in the same inflammasome complex. Recessive p.Ala160Thr mutation of NLRC4 determines an enhanced IL-1 and IL-18 cytokine release after stimulation with ATP or flagellin, which activate the NLRP3 and the NLRC4 inflammasome, respectively [120]. Moreover, the NLRC4 variant encoding p.Val341Ala, which results in gain of function, causes the SCAN4 syndrome, characterized by neonatal enterocolitis and autoinflammation with periodic fever and near-fatal or fatal episodes of autoinflammation [121]. In UC, therefore, unlike CD, inflammasomes’ gain of function mutations appear more likely to take place. The association between the inflammasomes and IBD is further supported by the involvement of NLRP6 and NLRP12, both exerting a protective role in experimental colitis [122,123]. In a recent case study, Tal et al. reported that a frameshift variant was detected in the NLRP12 gene in a patient with recurrent HSV-1 esophagitis and CD [124] and, as with NLRP1, NLRP12 was also found to regulate the commensal flora in animal models [114,123].

### 3.5. Inflammasomes Highlights in IBD

IBD are associated with an altered innate immunity through the de-regulated activity of inflammasomes, which might be activated or inhibited [109]. Mutations and polymorphisms of the NLR proteins NOD1/2, NLRC4, NLRP1 and NLRP3, determining loss of function with reduced inflammasome activity, are associated mainly with CD. Gain of function mutations or reduced expression of NLR proteins, such as NLRP12, which acts as a negative regulator of inflammasome activity, appears to be mainly correlated with UC (Figure 1). It would be reasonable to suggest various pathogenetic scenarios with differences between CD and UC: in CD a reduced, and in UC an excessively high inflammasome activity and innate immune response. The defective innate immune response in CD probably impairs clearance of luminal antigens and/or pathogens causing a selective pressure on bacterial species that, in turn, trigger chronic intestinal inflammation. Dysbiosis and bacterial derived metabolites might, in turn, activate NLRP3 and IL-18 release, the excessive production of which is implicated in enhancing gut inflammation by inducing the release of other cytokines, such as IL-22 and IL-17A, and in altering mucosal integrity by reducing the number of goblet cells and their maturation [119].

### 3.6. The Role of Laboratory Testing in the Evaluation of Inflammation in IBD

Generic marker of inflammation, such as complete blood count (CBC), C reactive protein (CRP) and Eritrocyte Sedimentation Rate (ESR) were widely used in the past for management of IBD patients [125], despite their low sensitivity and specificity [126]. Currently, it has been described an association between CRP levels and other CBC parameters with pseudopolyp formation in UC and fistulas in CD [127]. ESR has been described as useful for risk stratification model for defining severe ulcerative colitis, especially in pediatric patients, being correlated with both endoscopic and histologic activity of the colon in children with CD [128]. There is a wide consensus now on the fact that intestinal specific markers, such as fecal lactoferrin (fLact) and fecal calprotectin (fCal), have better utility than serum markers, their sensitivity and specificity in distinguishing IBD from function intestinal disorders being about 80% and above 80%, respectively [58,59,129,130].

## 4. Autoimmunity in IBD

The immune system is also involved in IBD extra-intestinal manifestations (EIMs) [131] and in the so-called “paradoxical” complications triggered by biological drugs, such as anti-TNF-α agents [132,133]. In various studies, it has been estimated that the occurrence of EIMs ranges from 21 to 36% in IBD patients [134], encompassing involvement of vascular and hematologic, genitourinary, cardiac, pulmonary, neurological, endocrine and metabolic systems, but more frequently, musculoskeletal, dermatologic, ocular and hepatobiliary systems, such as ankylosing spondylitis, erythema nodosum, uveitis and primary sclerosing cholangitis (PSC) [131,132,135].

Major groups of EIMs share features of autoimmune-related processes [135]. One of the proposed immunopathogenic model for UC is based on autoantigens shared by colon and different extracolonic organs—namely, human tropomyosin isoform 5, a cytoskeletal protein, and colon epithelial specific protein [134], triggering T cells activation, which helps priming a subset of B cells further expanding and producing IgG class 1 autoantibodies that can activate the complement system [135]. Genetic susceptibility is also recognized in the autoimmune mechanisms, as well as the probable role of molecular mimicry conducted by microbes [131]. Paradoxical complications are mainly immune-mediated inflammatory disorders, particularly skin lesions and joint inflammation, developed after administration of anti-TNF-α monoclonal antibodies, which resolve when discontinued [133]. Putative pathogenic mechanisms include an imbalance between inflammatory and regulatory cytokines that can favor an excessive immune response and induce autoimmunity [133]. Furthermore, anti-nuclear antibodies (ANA) and anti-double strand-DNA (anti-dsDNA) are frequently found in IBD patients, although rarely in association with a lupus-like syndrome, and several cases of vasculitis have been reported [133].

Moreover, population-based studies have registered a significantly more frequent incidence of immune-mediated diseases in IBD patients, including autoimmune hepatitis, celiac disease, atrophic gastritis, rheumatoid arthritis, type 1 diabetes and Grave’s disease, thus reinforcing the hypothesis of partially overlapping pathogenic mechanisms [136].

### Laboratory Testing in IBD: Autoimmunity Markers

Different autoantibodies have then been reported and studied in IBD, showing diagnostic value in differentiating UC and CD, and also a role in predicting disease course [137]. However, the most informative autoantibodies have proven to be anti-neutrophil cytoplasm antibodies (ANCA) and anti-Saccharomyces cerevisiae antibodies (ASCA) [138], although their pathogenic role has not been fully elucidated [137]. The first study demonstrating circulating autoantibodies in IBD appeared in 1959, when serum from children with UC caused immunoprecipitation of a γ-globulin that reacted against healthy human colonic tissue [139]. In a 2012 systematic review of autoantibodies in IBD, P-ANCA prevalence of was estimated at 6 to 38% of CD and 41 to 73% of UC, while ASCA were reported in 29 to 69% of CD and 0 to 29% of UC, with a sensitivity of 31 to 45% and a specificity of 90 to 100% had the best positive predictive value (PPV) for the diagnosis of IBD with respect to healthy subjects (97–100%) [137].

ANCA might be commonly directed against proteinase 3 (PR3) and myeloperoxidase (MPO), although it is believed that autoantibodies towards different antigens (e.g., elastase, cathepsin G, lactoferrin, lysozyme) also elicit a positivity in indirect immunofluorescence (IIF), which can return cytoplasmic or perinuclear patterns [140,141,142,143].

ANCA development has not yet been fully elucidated, although it has been suggested that there is involvement of the production of a complementary antigen to PR3 (cPR3) leading to anti-PR3 due to epitope spreading, or autoantibodies due to molecular mimicry following exposure to Staphylococcus aureus and Entamoeba histolytica, or even neutrophil apoptosis impairment that might increase exposure to autoantigens [144].

Although the clinical association for ANCA is mainly due to vasculitides [144] in IBD patients, P-ANCA, in particular, was initially found in patients with UC in 1961 [145], this finding being subsequently confirmed in several reports [138]. More recently, anti-PR3 antibodies also tested with chemiluminescent immunoassay (CIA) were associated to UC with an odds ratio (OR) vs. healthy controls equal to 12.8 and shown to differentiate UC from CD [146]. Therefore, the 2020 international consensus on ANCA testing prescribed that testing for ANCA be made only in selected cases of IBD, when the differential diagnosis between uncertain between CD and UC is uncertain, and by IIF, as the target antigens in IBD have not yet been well characterized [141].

ASCA, first reported in 1988 in CD [137,147], are directed against the cell wall mannan of Saccharomyces that share homology with intestinal bacteria and are commonly detected with ELISA assay [137,148]. Both IgG and IgA immunoglobulin classes can be found [138]. Different studies evaluating clinical performances of each class reported contradictory findings, possibly due to differences in the patient cohorts included, or analytical differences; however, a specificity and a positive predictive value (PPV) of up to 100% when comparing CD diagnosis versus UC or healthy subjects for simultaneous positivity of both ASCA IgG and IgA were reported, while best sensitivity of 71% was reported when either IgG or IgA class were found [149,150].

As only recently reported, together with ANCA and a panel of proteins including anti Escherichia coli outer membrane porin C (OmpC) and anti-flagellins antibodies, ASCA may be predictive of the development of CD within 5 years and of UC within 1 year [151].

Anti-laminaribioside carbohydrate IgG antibodies (ALCA), anti-chitobiosidecarbohydrate IgA antibodies (ACCA), anti-mannobioside carbohydrate IgG antibodies (AMCA), anti-laminarin IgA (anti-L) and anti-chitin IgA (anti-C) are novel antibodies against bacterial polysaccharides further associated with IBD [152,153], which is overall more prevalent in CD where reported PPVs versus UC range from 65 to 92% [137].

Further studies on IBD prediction are therefore needed to include ANCA and ASCA in validated clinical algorithms.

## 5. Concluding Remarks and Perspectives

A variety of molecular markers are being evaluated for IBD prevention, diagnosis and monitoring, as well as new therapeutic options that take into account this disease’s complex physiopathology, which has been further complicated by the recent COVID-19 pandemic. The SARS-CoV-2 entry receptor, angiotensin converting enzyme 2 receptor, is up-regulated in IBD and it is known that that SARS-CoV-2 infection involves the gastrointestinal tract, although IBD patients even under immunosuppressive treatment were not reported to have a worse COVID-19 disease nor a worsening IBD course [154].

Among the innovative approaches, proteomics studies have been successfully applied. As an example, in a recent study from our group, we found that fecal peptidome of IBD patients was highly enriched of low molecular weight peptides, in contrast to samples from individuals without gastrointestinal symptoms; in addition, peptidome was useful in distinguish UC from CD patients [18]. Intriguingly, analysis of the peptides-corresponding proteins showed that IBD stools are enriched in proteins involved in inflammation and immune response (data not shown). Another proteomic study from Leibovitzh et al. confirmed this finding, since serum C-X-C motif chemokine 9 (CXCL9) was identified as and early marker of CD, highly associated with the disease, reflecting the biological processes of immune and barrier dysfunction in IBD [155]. The metabolome has also been studied, highlighting the connection between IBD and microbiome pathway of differentially abundant molecules [156,157]. Although these are promising results, there is still an ongoing debate about whether metabolic findings are sufficient to explain the diseases complexity.

Overall, these studies depicted IBD as a complex disease, instigated and amplified by genetic susceptibility, environmental variables (exposome) and dysregulation of immune response, which finally perturb the immune–microbiome axis [158]. The current inefficiency in treatments suggest the correctness of the paradigm “one size does not fit all” in IBD; thus, personalized, tailored treatment for these diseases, based on individual tracts heterogeneity, could be advantageous [158]. Recently, the application of artificial intelligence (AI) methods has been used for facilitating the analysis, and for the integration and interpretation of large datasets. A recent meta-analysis in 2021 demonstrated that AI has been applied in 17 studies focused on IBD diagnosis and 5 studies focused on predicting risk of IBD [159]. Interestingly, colonoscopy and endoscopic images were used for generating AI model, with or without the addition of biochemical and molecular data and gene expression profiles [159], and very relevant results on sensitivity and specificity were found. In addition, some studies inspected the utility of AI in predicting disease severity [159]. Applications of AI include prediction analysis of potential molecular targets for drug discovery [160]. An example of this in the IBD setting is represented by the identification of a series of 17 potential drug targets among gene clusters grouped in the GO biological function term “response to stress”. Of particular interest is the Protein Kinase AMP-Activated Non-Catalytic Subunit Beta 1 (PRKAB1), because it is involved in epithelial dysfunction and in the process of epithelial to mesenchymal transition and is reported to be associated with IBD disease activity [160]. Finally, natural language processing tools (NLP), such as chatbots, have revealed valuable tools for improving user satisfaction and engagement of IBD patients [161]. Especially for patients’ monitoring, chatbots can improve patients’ self-management, empowerment and education [162]. However, strong guidelines are needed for reducing the risk of giving inappropriate (and maybe harmful) information.

## Figures and Tables

**Table 1:** Types of TLRs described as associated with CD and UC. CS: cell surface; IC: intracellular; Mø: Monocytes/macrophages; B: B lymphocytes; MCs: mast cells; N: neutrophils; DCs: dendritic cells; IECs: intestinal epithelial cells. Data reported in table were collected from [45,46,47].

TLR	Adapter	Compartments	Ligands	Cell Types	Main Alterations in IBD
TLR1	MyD88/TRIAP	CS	Di- and tri-acylated lipopeptides	Mø, B, MCs	No variation in IBD
TLR2	MyD88/TRIAP	CS	Bacterial lipoproteins or lipopeptides	Mø, B, MCs	Increased in active UC
TLR3	TRIF	IC	Double-stranded RNA (viral infection)	Mø, B, MCs, N, Myeloid DCs, IECs	Increased in active UC and CD
TLR4	MyD88/TRIAP, TRIF/TRAM	CS	LPS, free fatty acids	Mø, B, MCs, N, Myeloid DCs, IECs	Increased in UC and CD
TLR5	MyD88	CS	Bacterial flagellins	Mø, Myeloid DCs, IECs	Decreased in CD
TLR6	MyD88/TRAF6 and NF-κB pathway	CS	Di- and tri-acylated lipopeptides	Mø, B, MCs	Increased in UC
TLR7	MyD88	IC/Endosomal	Single stranded RNA (viral inflammation)	Mø	Increased in UC
TLR8	MyD88	IC/endosomal	RNA degradation products specific to microorganism (GU-rich single stranded RNA)	Mø, DCs	Increased in UC
TLR9	MyD88/TRAF6	IC	Nucleid acid	Mø, B, plasmacytoid DCs	Increased in UC

**Table 2:** Cytokines with a major role in IBD. Data reported in this table were obtained from [47,58,85,96].

Cytokine	Source of Secretion	Potential Function in Pathogenesis of Chronic Intestinal Inflammation in IBD
IFN alpha and IFN beta	DCs	Promote epithelial generation and induce IL-10 producing cells
IFN gamma	T cells and ILCs	Activate macrophages, augment antigen processing and induce epithelial cell death
TNF-alpha	Macrophages, DC and T cells	Pro-inflammatory action, pro-inflammatory cytokine production and angiogenesis, induce epithelial cell death, mediate T cell resistance against apoptosis and induce cachexia
IL-1	Neutrophils and macrophages	Pro-inflammatory actions: augment neutrophil recruitment, stimulate IL-6 production by macrophages, activate ILCs and promote tumor development. Significantly increased in UC patients
IL-6	Macrophages, fibroblasts and T cells	Perform pro-inflammatory action by means of IL-6 soluble receptor. Activate T cells and prevent apoptosis (via STAT3), induce macrophage activation, recruit immune cells, activate acute-phase proteins, induce epithelial cell proliferation
IL-10	T cells	Exert anti-inflammatory effects that inhibit both antigen presentation and subsequent release of pro-inflammatory cytokines, and induce STAT3 signaling in regulatory T cells
IL-12	Macrophages and DC	Induce Th1 cell differentiation via STAT4 activation in T cells, stimulate Th1-type cytokine production and activate ILCs; a link between innate and adaptive resistance
IL-13	T cells, mast cells, basophil and eosinophil and NKT cells	Induce intestinal epithelial cell alterations and barrier function; induce fibrosis
IL-17	Th17 cells and ILCs	Induce pro-inflammatory factors (including TNF-α, IL-6 and IL-1β) and anti-inflammatory effects in the mucosa; IL-17A exerts pro-fibrotic functions
IL-18	IECs	Act in synergy with IL-12 to promote the production of INF-g, causing severe intestinal inflammation
IL-21	Th1 cells	Induce production of TNF-α, IL-1, IL-6 and IL-8 in the mucosa, recruit neutrophils, induce secretion of matrix metalloproteinases by fibroblasts and favor tumor development
IL-22	T cells, ILC, neutrophils and DC	Exert a pro-inflammatory effect; increased in both CD and UC. Activate production of antimicrobial peptides by epithelial cells, induce proliferation of epithelial cells and favor tumor development via STAT3 activation
IL-23	Macrophages and DCs	Activate mucosal immune cells (e.g., T cells and macrophages) cells, augment TNF-α production and stabilize effector Th17 cell phenotype
IL-27	Macrophages	Exert pro-inflammatory effects by inducing T cell activation and Th1-type cytokine production and exert anti-inflammatory effects by blocking T cell expansion and inhibiting cytokine production by neutrophils
IL-33	Epithelial cells and myofibroblasts	Suppress Th1-type cytokine production and induce neutrophil influx

**Table 3:** NOD-like receptors (NLR) and IBD. The proteins, the belonging families, functions and associated diseases are reported. MHC: major histocompatibility complex; NOD: Nucleotide-binding oligomerization domain; VAMAS1: Vitiligo-associated multiple autoimmune disease 1; MSPC: Multiple self-healing palmoplantar carcinoma; JRRP: Congenital juvenile recurrent respiratory papillomatosis; FMF: Familial Mediterranean Fever; FCAS: Familial cold autoinflammatory syndrome; AIADK: Autoinflammation with arthritis and dyskeratosis; MWS: Muckle-Wells syndrome; RA: Rheumatoid arthritis.

Family	Protein	Gene	Mutation-Related Diseases	IBD-Associated	Function	Complex
NLR	MHC class II transactivator	*CIITA*	Bare lymphocyte syndrome RA	Unknown	Positive regulator of class II MHC	
NLRB	NRL family apoptosis inhibitory protein	*NAIP*	Spinal muscular atrophy	Unknown	Anti-apoptotic (inhibits CASP3, CASP7 and CASP9)	Sensor component of NLRC4 that recognizes and binds CprI from pathogenic bacteria *C. violaceum*
NLRC	NOD1	*NOD1*	IBD, Asthma, Behcet’s disease and sarcoidosis	Yes	Innate and adaptive immune responses and cellular homeostasis. Binds bacterial peptidoglycans, single-stranded RNA (ssRNA) from viruses and the metabolite sphingosine-1-phosphate	Interacts with RIPK2 activating NF-kB and MAPK signaling pathways
NLRC	NOD2	*NOD2*	Crohn’s disease and Blau syndrome	Yes	Innate and adaptive immune responses and cellular homeostasis. Binds LPS by recognizing the muramyl dipeptide (MDP), single-stranded RNA (ssRNA) from viruses and the metabolite sphingosine-1-phosphate	Interacts with RIPK2 activating NF-kB and MAPK signaling pathways. Interacts with NLRP1 leading to IL-1 release. Interacts with ATG16L1 leading to autophagy
NLRC	NOD-like receptor caspase recruitment domain containing proteins 3–5	*NLRC3*		Yes	Negative regulator of the innate immune response (negative regulation of NF-kB and type I interferon signaling pathways)	Prevents NLRP3 inflammasome formation and may affect NOD1- or NOD2-mediated NF-kB activation
		*NRLC4*	FCAS 4 Autoinflammation with infantile enterocolitis	Yes	Innate immune response. Promotes caspase-1 activation, cytokine production and macrophage pyroptosis	Homo-oligomerizes in the NLRC4 inflammasome and enters the NRLP3 inflammasome
		*NRLC5*	Pityriasis rubra pilaris Bare lymphocytic syndrome type I FMF	Unknown	Negative regulator of the innate immune response (negative regulation of NF-kB and type I interferon signaling pathways)	
NLRP	NACHT, LRR, and PRD containing proteins 1–14	*NLRP1-14*	NRLP1: VAMAS1 MSPC JRRP	Yes	NLRP1: Innate immunity and inflammation. Cytokines IL-1, IL-18 and gasdermin-D (GSDMD), leading to pyroptosis, an inflammatory form of programmed cell death	NRLP1 inflammasome response to various pathogen-associated signals, recruits pro-caspase-1 (proCASP1) and promotes caspase-1 (CASP1) activation; may be activated by MDP in a NOD2-dependent manner
			NRLP3: FCAS1 AIADK MWS CINCA syndrome	Yes	NLRP3: regulation of inflammation, immune response, and apoptosis. Stimulated by extracellular ATP, reactive oxygen species, K(+) efflux, crystals of monosodium urate or cholesterol, amyloid-beta fibers, environmental or industrial particles and nanoparticles, cytosolic dsRNA	NRLP3 inflammasome upstream activator of NF-kappaB signaling
			NLRP12: FCAS2	Yes	NLRP12 potent mitigator of inflammation Primarily expressed in dendritic cells and macrophages, inhibits both canonical and non-canonical NF-kB and ERK activation pathways Functions as a negative regulator of NOD2 by targeting it to degradation via the proteasome pathway. Promotes bacterial tolerance	
NLRX	NOD-like receptor with “unknown” domain	*NLRX1*	Histiocytic sarcoma Combined oxidative phosphorylation deficiency 4 Mooren Ulcer Mitochondrial Complex V Nuclear deficiency Type 3	Unknown	Regulator of mitochondrial antivirus responses. Promotes autophagy Enhances NF-kB and JUN N-terminal kinase dependent signaling through the production of reactive oxygen species. Regulates energy metabolism in a sex-dependent manner	Regulates NLRP3 inflammasome activation to attenuate apoptosis

Data from GeneCard^®^, The human gene database–https://www.genecards.org/ (accessed on 25 January 2023).
